# Ghrelin in Serum and Urine of Post-Partum Women with Gestational Diabetes Mellitus

**DOI:** 10.3390/ijms19103001

**Published:** 2018-10-01

**Authors:** Żaneta Kimber-Trojnar, Jolanta Patro-Małysza, Katarzyna E. Skórzyńska-Dziduszko, Jan Oleszczuk, Marcin Trojnar, Radzisław Mierzyński, Bożena Leszczyńska-Gorzelak

**Affiliations:** 1 Department of Obstetrics and Perinatology, Medical University of Lublin, Lublin 20-090, Poland; jolapatro@wp.pl (J.P.-M.); jan.oleszczuk@umlub.pl (J.O.); radekm1969@gmail.com (R.M.); b.leszczynska@umlub.pl (B.L.-G.); 2 Department of Human Physiology, Medical University of Lublin, Lublin 20-080, Poland; katarzyna.skorzynska-dziduszko@umlub.pl; 3 Department of Internal Medicine, Medical University of Lublin, Lublin 20-081, Poland; marcin.trojnar@umlub.pl

**Keywords:** gestational diabetes mellitus, ghrelin, bioelectrical impedance analysis, body composition, hydration status

## Abstract

Women with a previous history of gestational diabetes mellitus (GDM) have a significantly increased risk of developing type 2 diabetes, obesity, and cardiovascular diseases in the future. The aim of the study was to evaluate ghrelin concentrations in serum and urine in the GDM group in the early post-partum period, with reference to laboratory results, body composition, and hydration status. The study subjects were divided into two groups, that is, 28 healthy controls and 26 patients with diagnosed GDM. The maternal body composition and hydration status were evaluated by the bioelectrical impedance analysis (BIA) method. The concentrations of ghrelin in the maternal serum and urine were determined via enzyme-linked immunosorbent assay (ELISA). The laboratory and BIA results of the mothers with GDM were different from those without GDM. Urine ghrelin positively correlated with serum ghrelin and high-density lipoprotein cholesterol (HDL) levels in healthy mothers. There were direct correlations between urine ghrelin and HDL as well as triglycerides levels in the GDM group. Neither the lean tissue index nor body cell mass index were related to the serum ghrelin concentrations in this group. Only the urine ghrelin of healthy mothers correlated with the fat tissue index. Our results draw attention to urine as an easily available and appropriable biological material for further studies.

## 1. Introduction

Gestational diabetes mellitus (GDM) is one of the most common metabolic disorders of pregnancy and its incidence has increased by 10–100% in the last 20 years [1]. The exact worldwide prevalence of GDM remains unknown, as systematically synthesized data on this issue are lacking, and the only available information is that the prevalence of GDM largely differs among countries and may even be different in the regions of the same country, ranging from 0.6% to 15%, depending on the ethnicity and socio-economic status of the studied individuals [2]. Women with a previous history of GDM have a significantly increased risk of developing type 2 diabetes mellitus (T2DM), obesity, and cardiovascular diseases (CVD) in the future [3,4,5]. The risk of developing diabetes is 9.6 times greater for patients with GDM, with the cumulative risk being 25% 15 years post-diagnosis [6]. Identifying women with GDM as a high-risk group for subsequent diseases offers an opportunity to alter their future health. There is a huge need to use current research results regarding improved GDM management strategies, including primary prevention for the mothers who are at risk of developing subsequent complications [1,2].

In recent years, the importance of ghrelin as a significant risk factor for developing T2DM in patients with a history of GDM has been underlined [7]. Ghrelin is a 28 amino acid peptide that is mainly secreted in the stomach fundus cells, but is also produced, in smaller amounts, in other bodily organs, such as the hypothalamus, heart, pancreatic cells, lungs, adrenals, kidneys, and placenta [8,9,10]. Ghrelin is an orexigenic peptide that plays an important role in regulating disorders, such as insulin resistance, obesity, and diabetes [11]. Ghrelin increases food intake and causes weight gain, mainly in the fat tissue. It also controls energy metabolism, insulin secretion, inflammation, apoptosis, cardiovascular function, immune response, and neurodegeneration [12]. Along with the ghrelin role in the systemic metabolism, a variety of studies evaluated the therapeutic impact of ghrelin pathway modulation. While ghrelin agonism might offer the potential to treat diabetic gastroparesis and anorexia, associated with pathological underweight and cachexia, ghrelin receptor antagonism might be of therapeutic value to decrease body weight under certain conditions of obesity, and also to improve the glucose metabolism and T2DM [13]. The positive effect of exogenous ghrelin might be mediated through the protection of the endothelial cells by inhibiting proinflammatory cytokines [14]. The effects of ghrelin on atherogenesis might involve the lipid metabolism. Ghrelin signaling plays an important role in macrophage polarization and adipose tissue inflammation.

However, the correlations between ghrelin and lipid metabolism have been less studied. Similarly, in the literature, there is no clear evidence of any relationship between ghrelin levels and maternal anthropometry [15]. Furthermore, studies on urine ghrelin concentrations are limited [16,17,18]. As far as we know, its levels in urine have not been investigated in patients with GDM before.

The relationship between ghrelin and various biochemical and biophysical measurements in puerperal women with GDM still remains unknown. We hypothesized that the ghrelin concentrations in serum and urine would probably be impaired in the group of women with GDM in the early post-partum period, because of some disturbances, including changes in the body composition and hydration status.

## 2. Results

The comparative characteristics of the study groups, presented in Table 1, revealed that healthy women had significantly lower post-partum body mass index (BMI) and decreased levels of hemoglobin A1c (HgbA1c), fat tissue index (FTI), total body water (TBW), and extracellular water (ECW), as well as higher concentrations of albumin and high-density lipoprotein cholesterol (HDL).

No significant differences were observed between the groups in terms of other analyzed parameters (Table 1).

The urine ghrelin concentrations were positively correlated with the HDL levels in all of the patients, as well as after dividing the whole group according to GDM—both in the healthy and in the GDM groups. A significant positive correlation was also observed between the urine ghrelin and triglycerides levels, but only in the diabetic mothers (Table 2).

There was a direct correlation between the serum and urine ghrelin levels only in the healthy mothers (Table 2). Positive correlations were also found between the serum ghrelin concentrations and gestational BMI gain (ΔBMI 1) and BMI loss at 48 h after delivery (ΔBMI 2), with the exception of the GDM patients (Table 2). 

Negative correlations were found between the ghrelin levels (both in the serum and urine) and the post-partum BMI and ECW in the healthy mothers; intracellular water (ICW), lean tissue mass (LTM), and body cell mass (BCM) in all the studied patients as well as after dividing the whole group according to GDM—both in the healthy and GDM groups; TBW in all the patients, with the exception of the urine ghrelin concentrations in the studied GDM patients; and lean tissue index (LTI) and body cell mass index (BCMI) in all of the patients, with the exception of the serum ghrelin concentrations of the GDM patients (Table 2).

Moreover, the urine ghrelin concentration was negatively associated with FTI in the healthy mothers and ECW in the whole group (Table 2).

## 3. Discussion

This study demonstrated that lower levels of albumin and HDL, as well as higher HgbA1c concentrations, were present in the GDM mothers. These results are consistent with observations made by other authors. Women with a history of GDM exhibit altered CVD risk factors, including lower HDL concentrations, when compared with mothers with healthy pregnancies [19,20]. A previous meta-analysis found that GDM confers a seven-fold risk of future T2DM, and up to one-third of women with T2DM have been previously diagnosed with GDM [19]. Approximately 50% of women who are diagnosed with GDM will develop T2DM [1].

### 3.1. Significance of Urine and Bioelectrical Impedance Analysis (BIA)

The biological materials used in the study included serum and urine. A comparison of the results in these two types of biological fluids was performed, with the intention of finding a non-invasive diagnostic material. To the best of our knowledge, this study is the first to show urine ghrelin levels in women with GDM. 

The choice of urine as the studied material seems to be favorable with regard to the results of BIA, which was used to assess the maternal body composition and hydration status. BIA is a standardized technique, and is non-invasive and fast, and, therefore, it is well tolerated by patients [21,22,23]. The physical properties of BIA, its measurement variables, and their clinical significance have well been described in many previously published reports [22,24]. BIA appears to be a more accurate predictor of gestational and post-partum outcomes than BMI [25]. Pathological changes of maternal TBW using BIA measurements have been related to gestational maladaptation [26]. What is important in this context is that the BIA measurements must be interpreted considering the background of adequate reference values for the population of interest as bioelectrical properties, and their relationship to body composition is affected by height, weight, hydration status, and stage of life [26,27].

Very few studies concerning BIA in the assessment of GDM have been reported so far. Moreno Martinez et al. [28] observed that the body composition of women between 24 to 32 weeks of single gestation was different in the women with GDM than in women with normal glucose tolerance. Those women who had GDM showed a significant increase in the fat mass, with no significant changes in the fat free mass and TBW [28]. Our study results showed that mothers with GDM, when compared with the healthy controls, presented higher levels of not only FTI, which is defined as the adipose tissue mass divided by the square of the body height and is expressed in units of kg/m^2^, but also of TBW and ECW, where the latter consists of the interstitial water, plasma water, and transcellular water. In our study, we were able to find inverse correlations between ICW and ghrelin levels in the serum and urine in the whole study group, as well as in the subgroups, according to GDM—both in the healthy and GDM groups. In contrast to the findings in the healthy group, the GDM patients were characterized by ECW, which did not correlate with the serum and urine ghrelin concentrations, and by TBW, which, on the contrary, negatively correlated only with the serum ghrelin. The aforementioned results suggest that women in the early puerperium and having GDM present a certain degree of hydration status disturbances.

### 3.2. Associations between Ghrelin and BIA

A negative correlation between the total ghrelin level and fat cell volume needs to be highlighted [29,30,31]. Other studies reported finding inverse associations between the serum ghrelin levels, BMI, and total fat mass in women [30,31]. Our own study results revealed that ghrelin levels not only in the serum but also in the urine were negatively related to BMI, but only in the control group. Meanwhile, changes in pregnancy BMI (gestational BMI gain) and in the 48 h postpartum period (BMI loss at 48 h after delivery) were positively correlated to the level of ghrelin in the serum, but with the exception of patients with GDM, in whom no relationship was found between these parameters. We found a negative correlation between the urine ghrelin levels and FTI in the healthy patients, which is a novel finding, not previously reported. Ghrelin exists in a number of isoforms. In this study, only the total ghrelin was measured. A recent study aimed to clarify the relationship between the total, acyl, and des-acyl ghrelin molecules with insulin resistance. Total and des-acyl ghrelin were negatively associated with insulin resistance, whereas acyl ghrelin was positively associated with this metabolic abnormality [7]. Acyl ghrelin was directly correlated with BMI [14]. In previous studies, a positive correlation was found between the acyl ghrelin and body fat (expressed by FTI in the whole group), and inverse correlations were also observed between the acyl ghrelin and LTM in all of the patients and in the females group [14]. We observed a negative correlation between the ghrelin levels (both in the serum and urine) and LTM. However, LTI, which represents LTM divided by the square of the body height, was negatively correlated with the serum and urine ghrelin concentrations, with the exception of the GDM mothers’ serum. Moreover, in our study, the serum and urine ghrelin levels were inversely associated with BCM. In 1975, Emerson et al. [32] concluded that “fat storage in human pregnancy depends on food intake, as in the nonpregnant. BCM accumulation is independent of food intake, except protein, and depends on normal physiologic adjustments of pregnancy, which are upset by insulin lack in diabetes. The extra basal energy needs of gestation are determined by BCM acquisition, not total body weight” [32]. Therefore, taking into account the difference in BMI in the postpartum period between our patients, while there is no statistical difference in gestational weight gain, we introduced the BCMI coefficient, that is, the body cell mass divided by the square of the body height, because it seems to be a more precise parameter. This parameter negatively correlated with the serum and urine ghrelin concentrations, with the exception of the serum of the GDM group, similarly to the results of LTI.

### 3.3. Association of Ghrelin and the Nutritional Status

Ghrelin is able to influence the nutritional status in several mechanisms, which include the following: the regulation of fat distribution and energy metabolism in the muscle and liver, influence on inflammatory cytokines, and regulation of appetite. Numerous clinical studies confirmed that the ghrelin effect on nutritional status was related to BMI, muscle, and fat mass [14]. The findings of this study supported the concept that ghrelin affects the adaptive response to a caloric imbalance. Diabetic pregnancy may involve a positive or negative caloric balance. Circulating ghrelin decreases during physiological pregnancy, which represents a natural insulin-resistant state [8,33]. However, gestational glucose-induced ghrelin suppression is preserved at a lower level, and is probably not related to the degree of insulin resistance [34]. Few reports have evaluated plasma ghrelin concentrations in women with diabetes so far. Previous studies [7,35] reported lower plasma ghrelin concentrations in women with GDM. The persistence of this abnormality at 12 weeks of the post-partum period predicted maternal diabetes. Aydin et al. [36] found transitory low ghrelin levels in women with GDM, although the levels measured at two weeks after delivery were normalized. The same trend was observed in pregnant women with pre-gestational T2DM, although their ghrelin concentrations remained low in comparison to the control group. Our data showed that women with GDM had lower serum and higher urine ghrelin concentrations in the early post-partum period, in comparison with the healthy controls, that is, at 48 h after delivery, yet, these differences were statistically insignificant. On the other hand, there was a positive correlation between the serum and urine ghrelin levels, but only in the healthy mothers. This association between the ghrelin concentrations in these two biological materials was not observed in the GDM group. It seems that this irrelevant difference in favor of greater ghrelin levels in urine may be caused by disturbed metabolism of circulating ghrelin in the GDM mothers. This might have resulted from increased renal ghrelin clearance.

Gómez-Díaz et al. [8] observed that pregnant women with GDM and T2DM had significantly lower serum ghrelin levels compared with non-diabetic patients. This statistical difference can be connected to the fact that approximately half of the patients were receiving insulin in the study of Gómez-Díaz et al. [8], who in fact admitted that this treatment heterogeneity might have been a confounding factor. Meanwhile, all of the women with GDM in our study were treated with insulin during pregnancy until delivery, yet the serum and urine samples were taken 48 h after delivery, which means at least 48 h without gestational treatment. The design of our study included a 6-h fasting period prior to the collection of biological material and BIA analysis. It has been proven that ghrelin concentrations display a surge before meals, decline after meals, and then increase gradually until the next pre-prandial peak [10].

Moreover, our study was performed two days after delivery, which means two days after placenta delivery, whereas Gómez-Díaz et al. [8] evaluated the serum ghrelin levels in each mother during the last week before cesarean delivery. The ghrelin concentrations during pregnancy are partially related to the placental factors. The placenta plays an important role in maintaining appropriate circulating levels of maternal ghrelin during the later gestational stages. Therefore, diabetic pregnancy is a cause of endothelial dysfunction and premature placental aging, which may result in abnormal placental ghrelin secretion [8]. Notwithstanding, a ghrelin expression in the placenta has been shown to be increased in GDM [37]. Meanwhile, what is also extremely important is the time interval between the last insulin dose and the ghrelin level measurement. In our study, all of the patients with a GDM history received the last insulin injection at least 48 h earlier.

Our observations seem to confirm the results of Baykus et al. [38], who compared the serum concentrations of ghrelin in the period between weeks 24 and 28 of gestation to 24 h of the puerperium. The cited authors noted higher ghrelin levels in the post-partum period than during the healthy and GDM pregnancies. They concluded that this could explain the reduced gestational insulin resistance after delivery.

The circulating level of the total ghrelin is determined by the balance between the extent of secretion, degradation, and clearance. The clearance of circulating ghrelin includes the interaction with its receptor and excretion in the urine [31,39]. Ghrelin is mainly metabolized and excreted by the kidneys [14]. Many studies have suggested that changes in the circulating ghrelin levels reflect an individual’s nutritional status [40,41,42]. It was established that ghrelin is a negative regulator of insulin secretion, and the ghrelin signaling pathway plays an important role in glucose homeostasis [43,44]. These data identified the ghrelin–glucose–lipid metabolism interaction as a key homeostatic process modulating energy balance [44].

### 3.4. Associations between Ghrelin and Maternal Laboratory Results

Our findings revealed that the urine ghrelin concentrations correlated positively with the serum ghrelin and HDL levels in the healthy puerperal mothers. In contrast to these findings, there was no correlation between the urine and serum ghrelin in the women with GDM, with normal results of creatinine and glomerular filtration rate (GFR) in this group taken into account. Furthermore, the urine ghrelin levels in the diabetic group correlated not only with HDL, but also with the triglycerides levels. Meanwhile, in the previous study, the serum ghrelin correlated inversely with the fasting blood glucose, HgbA1c, and urine albumin-to-creatinine ratio, but exhibited a positive correlation with HDL only in the middle-aged (41–64 years) and old (65–76 years) subjects with newly diagnosed T2DM and normal glucose tolerance [44]. Similar results were found in premenopausal women with the metabolic syndrome, where the serum ghrelin negatively correlated with triglycerides, fasting blood glucose, and the homeostasis model assessment-estimated insulin resistance (HOMA-IR) index, however, it correlated positively with HDL [45]. It is worth paying attention to the fact that HDL represents approximately 20% of the total plasma cholesterol and is inversely related to the occurrence of CVD. In women without GDM, HDL levels decrease during the initial 12 weeks post-partum, but the magnitude of this decline is small. Moreover, it has been shown that during pregnancy, women with GDM have lower levels of HDL than those without GDM [46].

Our study is probably the first to report that the urine ghrelin represents a potential biomarker for the future use in women with GDM. Urine as a material for the assessment of ghrelin concentrations in human studies has been used in patients with epilepsy, stroke, urinary tract infections, and nephrotic syndrome [9,16,17,18]. Taskin et al. [16] found no statistically significant difference between the serum, urine, and saliva levels of ghrelin in children with the newly diagnosed idiopathic generalized epilepsy. The authors concluded that bodily fluids such as urine and saliva might be used to determine ghrelin levels [16].

In the literature, there are reports on the results of the comparison of ghrelin concentrations in diabetic mother’s serum and breast milk. Aydin et al. [47] showed that two days after delivery, the women with GDM and pre-gestational diabetes mellitus (PGDM) had more than two-fold lower colostrum and serum levels of ghrelin than the lactating non-diabetic women. Fifteen days after delivery, however, the GDM and healthy groups showed similar levels of ghrelin in the mature milk and serum. The results of Aydin et al. [47] indicate that the mothers with GDM had a significant decrease in the serum and colostrum ghrelin levels. The authors emphasized that this was a temporary effect lasting only until the early puerperium (two days after delivery). This peptide hormone is completely restored and returns to normal levels after 15 days of puerperium, but not in the case of women with PGDM.

According to our knowledge, there is no reported study investigating and comparing results in the serum and urine obtained in the early post-partum period in patients with and without GDM; thus, we consider this to be an innovative study in this regard. However, there are some limitations to the current study. Firstly, the sample size was small, so the conclusions may not be definitive. Also, we did not collect data regarding acyl ghrelin, although both acyl and des-acyl ghrelin levels are altered by diabetes [8]. Finally, we cannot compare the ghrelin levels to those in other studies, because of the heterogeneity of the measurement methodology, as well as the lack of availability of such results.

## 4. Materials and Methods

The study comprised women who were in a singleton term pregnancy (after 37 weeks of gestation) and were hospitalized at the Chair and Department of Obstetrics and Perinatology, at the Medical University of Lublin. The data collection was performed between March 2016 and February 2017. All of the study subjects included in this study were Caucasian and they were divided into two groups, namely: The first being 28 healthy controls—women without any metabolic disorders, with normal three results of the 2-h-75 g-oral glucose tolerance test (OGTT) at 24–28 weeks of gestation. Characteristics of this subgroup also included no concomitant diseases, only vitamin-iron supplementation, normal pre-pregnancy BMI (i.e., between 18.5 and 24.99 kg/m^2^), normal gestational weight gain (i.e., 11.5–16 kg) [48], and proper gestational age. The second group consisted of 26 patients with diagnosed GDM, on a diet, and receiving insulin treatment. Of the subjects, 60% of those with GDM were treated by the intensive insulin therapy and 40% of them were controlled with only one basal insulin injection per day. The diagnostic criteria for GDM were based on the OGTT at 24–28 weeks of gestation, as follows: fasting glucose ≥5.1 mmol/L (92 mg/dL), or one hour plasma glucose result of ≥10.0 mmol/L (180 mg/dL), or a two-hour plasma glucose result of ≥8.5 mmol/L (153 mg/dL) [49,50].

The exclusion criteria from the study comprised the following: multiple pregnancy, insufficient and excessive gestational weight gain, chronic infectious diseases, current urinary infections, abnormal laboratory results (e.g., the complete blood count, creatinine, GFR findings), metabolic disorders (such as polycystic ovarian syndrome; except those listed in the inclusion criteria for the studied groups), mental illness, cancer, liver diseases, cardiovascular disorders, fetal malformation, premature membrane rupture, intrauterine growth retardation, the presence of metallic prostheses, and pacemakers or cardioverter-defibrillators.

Anthropometric measurements and sampling were performed after a 6-h fasting in the early post-partum period (i.e., 48 h after delivery). The maternal body composition and hydration status were evaluated by means of the BIA method and with the use of a body composition monitor (BCM) (Fresenius Medical Care). The serum levels of albumin, HgbA1c, and lipid profile were measured by a certified laboratory. After centrifugation, all of the collected maternal serum and urine samples were stored at –80 °C. The concentrations of ghrelin in these materials were determined using commercially available kits and in compliance with the manufacturer’s instructions (Wuhan EIAab Science Co., Wuhan, China) via traditional enzyme-linked immunosorbent assay (ELISA). The survey was performed in duplicates for each patient.

All of the patients were informed about the study protocol, and detailed written consent was obtained from each patient who agreed to participate in the study.

The study protocol received approval from the Bioethics Committee of the Medical University of Lublin (no. KE-0254/221/2015 (25 June 2015) and no. KE-0254/348/2016 (15 December 2016)).

All of the values were reported as the median (interquartile range 25–75%). The differences between the groups were tested for significance using Mann–Whitney *U-*test. The Spearman’s coefficient test was used for the correlation analyses. All of the analyses were performed using the Statistical Package for the Social Sciences software (version 19; SPSS Inc., Chicago, IL, USA). A *p*-value of <0.05 was considered statistically significant.

## 5. Conclusions

The results of our study revealed differences in the laboratory results between the mothers with and without GDM at 48 h after delivery (i.e., albumin, HgbA1c, and HDL levels), as well as in the body composition (FTI) and hydration status (TBW and ECW). The urine ghrelin levels correlated positively with the serum ghrelin and HDL levels in the healthy puerperal mothers. In contrast to these findings, there was no correlation between the urine and plasma ghrelin concentrations in the GDM women. In this group, the urine ghrelin levels were associated not only with HDL, but also with triglycerides levels. 

In all of the studied patients, the serum and urine ghrelin levels negatively correlated with ICW and BCM. TBW was not associated with the urine ghrelin of diabetic mothers. Neither LTI nor BCMI were related to the serum ghrelin concentrations in this group. Only the urine ghrelin of the healthy mothers correlated with FTI.

Our results draw attention to the possibility of using urine as an easily available and appropriable biological material in further studies on GDM. It seems that the consideration of the evaluation of ghrelin in the urine should be taken into account in future studies, especially regarding anthropometry, body composition, and hydration status. Attention should be paid to the high statistical significance of the correlations obtained between the results of the urinary ghrelin in healthy patients with their body composition and hydration parameters.

The physiological and pathological significance of these findings requires further elucidation.

## Figures and Tables

**Table 1:** Comparison of characteristics of the subjects.

Variables	Control Group (*n* = 28)	GDM Group (*n* = 26)	*p*
**Day of Delivery**			
fasting blood glucose (mg/dL)	83.5 (73.0–91.0)	85.0 (82.0–98.0)	0.107
gestational weight gain (kg)	15.0 (8.0–15.6)	13.3 (9.2–15.0)	0.086
ΔBMI 1 (kg/m^2^)	5.4 (2.97–5.6)	5.08 (3.11–5.72)	0.085
**2nd Day of Post-Partum Period**			
BMI (kg/m^2^)	22.0 (21.0–23.9)	28.8 (25.3–30.65)	0.0011 *
ΔBMI 2 (kg/m^2^)	2.49 (2.08–4.16)	2.2 (2.11–2.7)	0.306
hemoglobin A1c (%)	5.3 (4.6–5.4)	5.5 (5.2–5.6)	0.018 *
albumin (g/dL)	3.68 (3.43–3.73)	3.46 (3.37–3.64)	0.007 *
total cholesterol (mg/dL)	249.0 (188.0–287.0)	209.0 (192.5–247.5)	0.217
HDL (mg/dL)	78.0 (75.0–82.0)	67.5 (54.5–73.5)	0.0013 *
LDL (mg/dL)	129 (93–152)	107 (85.5–129)	0.146
triglycerides (mg/dL)	177 (150–254)	240.5 (170–261)	0.109
serum ghrelin (ng/mL)	0.933 (0.646–1.115)	0.395 (0.19–1.226)	0.116
urine ghrelin (ng/mL)	0.102 (0.096–0.288)	0.212 (0.067–0.598)	0.225
total body water (L)	30.1 (25.2–35.0)	33.8 (31.1–35.6)	0.0015 *
extracellular water (L)	14.9 (13.0–15.7)	16.3 (15.0–17.2)	0.00014 **
intracellular water (L)	15.7 (13.5–17.8)	17.3 (15.8–17.8)	0.051
lean tissue mass (kg)	30.0 (27.0–37.2)	31.7 (30.8–33.1)	0.79
lean tissue index (kg/m^2^)	10.1 (9.4–13.1)	11.8 (11.0–12.5)	0.073
fat tissue index (kg/m^2^)	10.1 (9.1–13.8)	15.1 (13.3–17.9)	0.0012 *
body cell mass (kg)	15.0 (12.8–20.1)	17.1 (15.5–17.7)	0.454
BCMI (kg/m^2^)	5.31 (4.8–7.17)	6.19 (5.46–6.63)	0.085

The results are shown as the median (interquartile range 25–75%). A *p* value of <0.05 was considered significant. * *p* < 0.05; ** *p* < 0.001; BCMI—body cell mass index; BMI—body mass index at 48 h after delivery; ΔBMI 1—gestational BMI gain; ΔBMI 2—BMI loss at 48 h after delivery; HDL—high-density lipoprotein cholesterol; LDL—low-density lipoprotein cholesterol; GDM—gestational diabetes mellitus.

**Table 2:** Correlations between the maternal serum ghrelin levels and urine ghrelin levels and the selected parameters.

Variables	All the Studied Women		Healthy Group		GDM Group	
	Serum Ghrelin	Urine Ghrelin	Serum Ghrelin	Urine Ghrelin	Serum Ghrelin	Urine Ghrelin
ΔBMI 1	0.313 *	−0.296 *	0.6 *	−0.028	0.145	0.285
BMI	−0.196	−0.181	−0.428 *	−0.771 ***	−0.394	−0.018
ΔBMI 2	0.368 *	−0.048	0.543 *	−0.028	0.331	0.284
hemoglobin A1c	0.022	−0.199	0.319	−0.377	0.115	−0.303
albumin	−0.012	0.079	−0.086	−0.086	−0.036	0.1
total cholesterol	−0.011	0.089	−0.202	0.001	0.224	0.212
HDL	−0.061	0.312 *	0.145	0.696 **	−0.115	0.491 *
LDL	0.025	−0.073	−0.257	−0.086	0.079	−0.358
triglycerides	0.133	0.081	0.319	−0.203	0.333	0.564 *
urine ghrelin	0.116	-	0.543 *	-	0.136	-
total body water	−0.241*	−0.374 **	−0.429 *	−0.943 ***	−0.427 *	−0.264
extracellular water	−0.188	−0.239 *	−0.486 *	−0.886 ***	−0.409	−0.200
intracellular water	−0.276 *	−0.377 *	−0.429 *	−0.714 **	−0.630 *	−0.493 *
lean tissue mass	−0.288 *	−0.443 **	−0.486 *	−0.829 ***	−0.600 *	−0.509 *
lean tissue index	−0.301 *	−0.452 ***	−0.486 *	−0.829 ***	−0.287	−0.451 *
fat tissue index	−0.042	0.034	0.257	−0.429 *	−0.355	0.045
body cell mass	−0.294 *	−0.463 ***	−0.486 *	−0.829 ***	−0.442 *	−0.619 *
BCMI	−0.53 **	−0.534 ***	−0.428 *	−0.943 ***	−0.29	−0.509 *

Statistically significant values are given in the bold type. * *p* < 0.05; ** *p* < 0.001; *** *p* < 0.0001. BCMI—body cell mass index; BMI—body mass index at 48 h after delivery; ΔBMI 1—gestational BMI gain; ΔBMI 2—BMI loss at 48 h after delivery; HDL—high-density lipoprotein cholesterol; LDL—low-density lipoprotein cholesterol.
